# Association between red blood cell distribution width and the prognosis of brain death in patients with a Glasgow Coma Scale < 6

**DOI:** 10.1038/s41598-023-39836-6

**Published:** 2023-08-28

**Authors:** Marzieh Latifi, Habib Rahban, Elahe Pourhosein, Daniel Shostak, Sanaz Dehghani

**Affiliations:** 1 Medical Ethics and Law Research Center, Shaheed Beheshti University of Medical Sciences, Tehran, Iran; 2 Cardiovascular Research Foundation of Southern California, Beverly Hills, CA USA; 3 Southern California Medical Education Consortium, Temecula Valley Hospital, Universal Health System, Temecula, CA USA; 4 Sina Organ Procurement Unit, Sina Hospital, Tehran University of Medical Sciences, Hassan-Abad Sq. Emam Khomeini St., Tehran, 1136746911 Iran; 5 Iranian Tissue Bank and Research Center, Tehran University of Medical Sciences, Tehran, Iran

**Keywords:** Biomarkers, Medical research

## Abstract

Red blood cell distribution width (RDW) has been reported as a meaningful prognostic factor in various diseases. Our study compared patients’ RDW levels and prognosis at admission and discharge time. A total of 128 patients 77 patients who suffered brain death (subject group), and 51 patients who were discharged from the hospital (control group) with GCS ≤ 6 were recruited from 60 hospitals for this study. Demographical data and RDW measurements in these patients at admission time and brain death/discharge time were extracted into two groups. 46 (35.9%) patients were females and 82 patients (64.1%) were males with a median age of 36 years old. A significant difference in baseline characteristics of GCS (*P* < 0.001), RDW at admission time (*P* < 0.001), and RDW at discharge or brain death time (*P* < 0.001) were noted between the two groups. In the overall population, RDW at admission time had a median value of 13.75% and was positively correlated with gender (*P* < 0.04, rs = 0.582) and age (*P* < 0.023, rs = − 0.201). Initially, there were no significant differences in RDW upon admission. However, upon discharge, although the RDW in the control group was not significant (*P* < 0. 1), the RDW level at the time of brain death was notably 0.45 fold higher (*P* = 0.001) compared to the time of admission. The standardized residuals at the two-time points showed an approximately normal distribution. The most effective RDW cut-off in Brain death was determined as 14.55. Based on the findings, using RDW as a prognostic factor has a sensitivity of 0.468 and a specificity of 0.137 in diagnosing brain death. RDW biomarker is a simple and inexpensive laboratory test that may be seen as a valuable perspective for initial patient evaluation. RDW is a powerful marker for the prognosis of brain death in patients with a GCS ≤ 6 at admission time, in order to identify a subset of patients who may require more aggressive management in the trauma center.

## Introduction

Brain death is a medico-legal term that describes the irreversible loss of function of the brainstem and the brain in whole. Brain death can mostly be diagnosed clinically and/or with an apnea test^1^. Early diagnosis of brain death is important to expedite organ transplantation, provide closure for loved ones, and prevent unnecessary negative medical interventions and when possible expedite organ transplantation^2^.

Brain death leads to noticeable hemodynamic, hormonal, and metabolic changes that, if untreated, may result in cardiac arrest^3^. Assessing risk factors correlating with mortality can help improve the quality of organs^4^.

A hematologic index automatically calculated by blood cell counters is red blood cell distribution (RDW)^5^. This parameter of variability in the size of circulating erythrocytes is called an RDW^6^. Elevated RDW levels can result from any disease process that causes the premature release of reticulocytes into the circulation. Associated with elevated inflammatory markers and RDW have been reported in several studies^7,8^.

Recently, RDW was found to be a strong independent prognostic factor for several different pathologies such as traumatic brain injury (TBI), malignancies^5^, heart diseases^9–11^, ischemic cerebrovascular disease^12^, and cerebrovascular accident^13^.

According to Lorente, RDW was a prognostic marker for TBI with correlations between RDW and mortality^14^. Information about the value of RDW as a predictor of brain death is more limited. The purpose of this study was to determine whether brain death in patients with Glasgow Coma Scale < 6 (GCS ≤ 6) can be predicted via red blood cell distribution width (RDW).

## Methods and materials

This was a multicenter retrospective case-control study that consecutively enrolled a series of patients with GCS ≤ 6 who were admitted to 60 hospitals affiliated with two main organ procurement units (OPUs) in Tehran University of medical sciences between Jan 1st, 2019 and March 31st, 2022.

Of the 597 study patients, only 128 patients met the study criteria. 77(60.16%) patients with GCS ≤ 6 suffered brain death were placed into the subject group (Brain-dead group) and the remaining 51 (39.84%) patients were discharged from the hospital after recovery were placed into the control group (non-brain-dead group).

Brain death patients were detected by a GCS of 3 and the progressive absence of at least three out of six brain stem reflexes or a FOUR score of E0M0B0R0.

Same with the subject group, all control groups met the following criteria at the time of this survey. GCS at admission time was ≤ 6, there was no history of taking blood transfusion (RBC, Plasma) before hematological testing, and any illness possibly affecting RDW levels such as anemia, thalassemia trait, hereditary elliptocytosis, hemoglobin C disease, hypertension, diabetes, heart disease, inflammatory bowel disease (IBD), Pulmonary disease (PD), cerebrovascular diseases, as well as in hypertensive patients^15^, there was no history Hepatitis C and B Antigen Positive^3^, and all patients who intake any drugs which can effect on RDW were excluded to this study. In addition, RDW values must be checked in admission time and discharge/brain death time).

Detailed demographics, RDW as clinical, and laboratory data, GCS scores were measured on admission and prior to hospital discharge in the control group and at admission time, the time of brain death for the subject group using an automated cell counter (Sysmex Poch-100iV Diff).

RDW was determined by automated complete blood cell counts from a baseline blood sample processed at each site’s clinical laboratory or the most recent results available in patients lacking a baseline sample. All data were retrieved from medical records.

This study conducted according to declaration of Helsinki as a statement of ethical principles for medical research. Written informed consent be obtained for the publication of information from donor’s families (for brain death group) and control group. This study was approved by the ethics committee (IR.TUMS.IKHC.REC.1400.020), Tehran University of Medical Sciences).

### Statistical analysis

After evaluating the normality of the data using the Shapiro–Wilk test, descriptive statistics (frequency, mean and standard deviation) were studied. Changes in RDW at different time points were evaluated using a paired T-test. The strength of the association between RDW and clinical variables was assessed by univariate linear regression. An independent sample t-test was performed to compare the results between the study subjects and the controls. The mean changes of RDW level among the three groups were assessed with repeated measure analysis of variance. The receiver operating curve was used to determine the validity of different parameters in separating cases with RA from controls and the area under the curve (AUC). Meanwhile, the sensitivity, specificity, and cut-off values were calculated. In all analyses, a significance level of less than 0.05 was considered in SPSS16.

### Ethical approval

Ethical approval to report this case was obtained from * Tehran University of Medical Sciences (IR.TUMS.IKHC.REC.1400.020) *.

### Participation informed consent

Informed consent was obtained from all subjects’ legal guardians to participate in this research. NO organs/tissues were procured from prisoners.

### Statement of informed consent

Written informed consent was obtained from a legally authorized representative(s) for anonymized patient information to be published in this article.

## Results

According to the obtained results, the mean age of the participants was 31.58 ± 15.54 (median age: 30 years). In addition, 82 (64.1%) of the participants were male, and 60 (46.68%) of the cause of brain death was head trauma. The mean and the median of the demographic data are shown in Table 1.

**Table 1:** Baseline characteristics of study population.

Variable		All (n = 128)	Subject group (n = 77)	Control group (n = 51)	*P* value
Gender	Female	46 (35.9)	31 (40.3%)	15 (29.4)	0.14
	Male	82 (64.1)	46 (59.7%)	36 (70.6)	
Blood group	O	38 (29.9)	23 (29.9%)	15 (30)	0.76
	A	44 (34.6)	28 (36.4%)	16 (32)	
	B	36 (28.3)	22 (28.5%)	14 (28)	
	AB	10 (7.2)	4 (5.2%)	6 (10)	
Cause of problem	Trauma	60 (46.68)	39 (49.5)	21 (43.13)	0.33
	Toxicity	8 (6.25)	5 (6.5)	3 (5.88)	
	Ischemic CVA	24 (18.75)	13 (16.9)	11 (21.56)	
	Post CPR	11 (8.59)	6 (7.8)	5 (9.8)	
	Tumor	5 (3.9)	3 (3.9)	2 (3.9)	
	Other	20 (15.62)	11 (14.3)	9 (17.6)	

Variable	Mean ± SD (Median)	Mean ± SD (Median)	Mean ± SD (Median)	*P* value	
Age	31.58 ± 15.54 (30)	29.07 ± 15.77 (27)	35.93 ± 14.36 (36)	0.054	
GCS	4.33 ± 1.99 (4)	4.33 ± 2.6 (3)	4.33 ± 0.73 (4)	0.001	

Additionally, 5.27 ± 4.07 days was the time interval between admission and brain death confirmation. The main characteristics of the study samples are reflected in Table 1.

A significant difference in baseline characteristics of GCS (*P* < 0.001), RDW at admission time (*P* < 0.001), and RDW at discharge or brain death time (*P* < 0.001) were noted between the two groups. (Tables 1, 2).

**Table 2:** The level of RDW in interval study period.

Variable	All (n = 128)	Subject group (n = 77)	Control group (n = 51)	*P* value
	Mean & SD	Mean & SD	Mean & SD	
RDW at admission time	13.92 ± 2.04	14.46 ± 1.89	13.38 ± 2.33	0.001
RDW at discharge/brain death	14.86 ± 2.14	16.36 ± 1.92	13.76 ± 2.12	0.001

Whereas there is no significant difference between gender (*P* = 0. 14), blood group (*P* = 0. 76), age (*P* = 0.054) and cause of problem were noted between the two groups (*P* = 0.33) (Table 1).

Furthermore, we also divided the cause of brain death into traumatic and non-traumatic groups in the subject group. An ANOVA test revealed that RDW levels at admission time and at the time of brain death were consistently higher in the traumatic patients' group compared to the non-traumatic ones (*P* = 0.008).

Based on Table 2, the RDW levels were constantly increasing throughout the study.

There were no significant differences between RDW at admission time (13.38 ± 2.33) and RDW at discharge time (13.76 ± 2.12) in the control group (*P* = 0.1).

**Figure 1:**
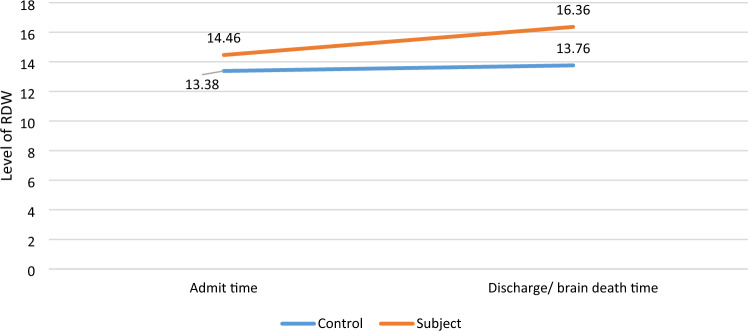
RDW values at admission time and time of brain death in the subject group compared to RDW values at admission time and time of discharge in the control group.

In contrast, RDW level at the time of brain death (16.36 ± 1.92) was higher compared to the time of admission (14.46 ± 1.89). The standardized residuals at the two-time points showed an approximately normal distribution (reflected in Fig. 1).

In the overall population, RDW at admission time had a median value of 13.7% (IQR: 13.92 ± 2.04) and was correlated with gender (*P* < 0.04, rs = 0.582), age (*P* < 0.023, rs = − 0.201), blood group (*P* < 0.54, rs = 0.54), and cause of brain death (*P* < 0.6, rs = 0.47).

After relevant confounder adjustment using the univariate regression model, RDW value (OR 1.475, 95% confidence interval 1.16–1.86, *P* = 0.001) was an independent variable for brain death. Participants with RDW in the highest quartile had a fully adjusted hazard ratio for brain death compared with those with RDW in the lowest quartile. Higher levels of RDW at admission time were also associated with an increased risk of brain death (*P* = 0.007).

**Figure 2:**
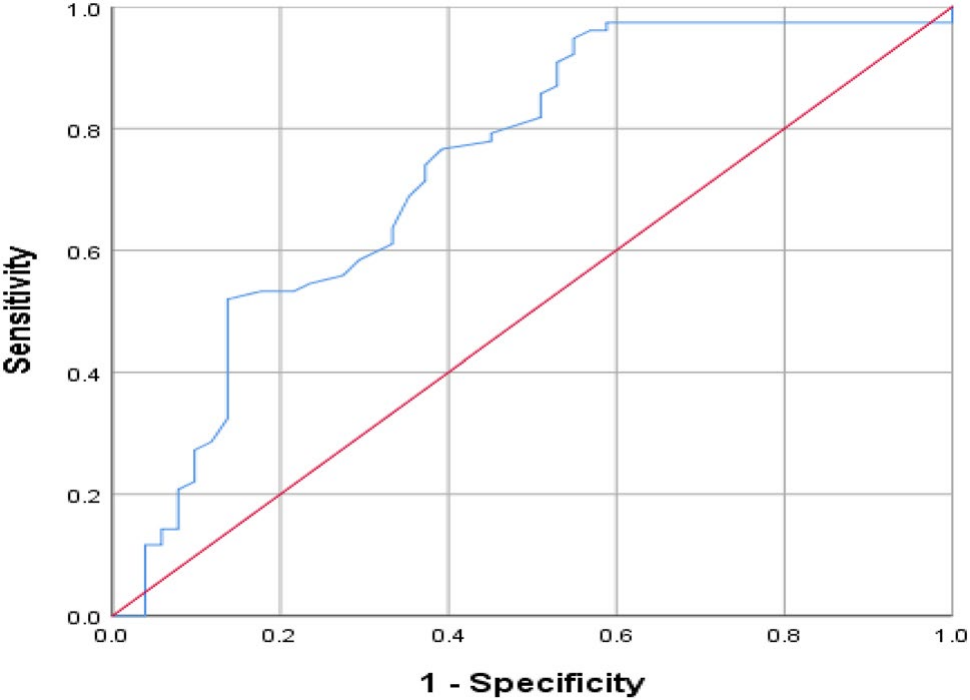
The relative operating characteristic (ROC) curve of RDW values for predicting brain death.

ROC curves of RDW levels were used to identify brain-dead group with a statistically significant level (area under the curve of 0.737; 95% confidence interval [CI], 0.645–0.829). As shown in Fig. 2, the best cut-off level for RDW in Brain death was 14.55. RDW in the diagnosis of brain death prognosis had a sensitivity of 0.468 and a specificity of 0.137.

## Discussion

Previous studies explored the elevations in RDW levels associated with elevated inflammatory markers^16^, Previous studies explored the relationship between RDW and prognosis in patients with breast cancer^17^, Multiple Sclerosis (MS)^18^, and coronary artery disease^19^.

The role of RDW as a prognostic factor for brain death patients with GCS ≤ 6 is unclear.

Our study of the patients with GCS ≤ 6 showed a meaningful RDW increase in the brain death group compared to non-brain-dead patients.

The cut-off of 14.55% in the ROC curve demonstrated that RDW is a specific and sensitive biomarker for diagnosing brain death. According to our results, RDW may be a prognostic factor for brain death in patients with GCS < 6.

New markers with prognostic features can help to identify vulnerable patients and optimize patient care and management in high-risk groups^20^. Based on our results more attention should be paid to patients with a higher RDW at admission time.

To the best of our knowledge, there have been few studies investigating RDW as a prognostic factor of brain death. We were able to locate only one study by Nevzat Mehmet that identified RDW as a predictor of brain death. The study documented that RDW levels on the days of brain death and cardiac arrest were significantly higher than on the day of admission^21^.

Consist of our results, Lippi et al. showed significant RDW increase levels in traumatic patients upon admission compared to others. According to their findings, patients who had experienced trauma were three times more likely to have higher RDW values than those who had not experienced trauma^22^.

Zhang et al.^23^, showed that RDW might be a reliable prognostic biomarker for Traumatic brain injury mortality.

Similar to our results, Lee et al.^24^ revealed that RDW can independently predict mortality in trauma patients.

On the contrary, Sadaka et al. demonstrated that RDW was a poor prognostic factor of mortality with an AUC value of 0.66 in traumatic brain injury patients^16^.

Results have shown a correlation between RDW, gender, and age. Like our finding, Hoffmann et al.^25^ found a strong association between RDW and age; however, they didn’t find any association between RDW and gender.

## Conclusion

In a retrospective study of 162 patients, we found that RDW is a powerful marker for the prognosis of brain death, in order to identify a subset of patients who may require more aggressive management in the trauma center^22^. In cases with loss of consciousness due to head injury, a high level of RDW could be associated with a higher risk of brain death. Therefore, RDW is a simple and inexpensive biomarker that could be seen as a valuable perspective for initial patient evaluation^22^. Utilizing these findings could aid in identifying the patients who would benefit from more aggressive management.

Considering the study’s novelty, there was no similar research data to reference, resulting in some limitations in the study. It was a retrospective study with a small number of patients in both groups. Some patients were excluded from the study, given the abovementioned inclusion criteria. To improve future studies, we recommend increasing the number of patients and expanding the inclusion criteria, such as GCS to > 6.

The strength of this research was the distribution of all hospitals in Iran. Therefore, our results may be applicable to other organizations with different patient populations and larger sample sizes with a wider variety of socio-demographic characteristics. Clearly, more studies are needed to confirm the validity of the present results in a larger population.

## Data Availability

The datasets used and/or analyzed during this research study are available from the corresponding author (Dr. Sanaz Dehghani with email: sanaz_dehghani2002@yahoo.com) on reasonable request.
